# Real-world experience of hereditary angioedema (HAE) in Mexico: A mixed-methods approach to describe epidemiology, diagnosis, and treatment patterns

**DOI:** 10.1016/j.waojou.2023.100812

**Published:** 2023-09-13

**Authors:** Sandra Nieto, Ileana Madrigal, Francisco Contreras, María Eugenia Vargas

**Affiliations:** a Specialty in Pediatrics and Pediatric Hematology. Genetics of Nutrition Unit, Instituto Nacional de Pediatría, CDMX, Mexico; b Specialty in Allergy and Immunology. High Specialty Medical Unit (UMAE), Centro Médico Nacional de Occidente, IMSS, Guadalajara, Mexico; c Specialty in Pediatrics and Allergy and Clinical Immunology. Allergy and Immunology Department, Instituto Nacional de Pediatría, CDMX, Mexico; d Specialty in Internal Medicine, Allergy and Clinical Immunology Master's Degree in History. Allergy and Immunology Department, Centro Médico Nacional 20 de Noviembre, ISSSTE, CDMX, Mexico

**Keywords:** Hereditary angioedema, Prevalence rate, Real-world experience, Treatment

## Abstract

**Introduction and objectives:**

Due to the lack of structured and systematic information available, the aim of this study was to describe the epidemiology, diagnosis, healthcare processes, and treatment patterns of hereditary angioedema (HAE) in Mexico. To achieve this, different data sources were consulted regarding medical literature, structured health system databases, and angioedema-specialized physicians (AEP) opinion regarding HAE.

**Material and methods:**

A mixed methods approach was conducted in 4 phases: I) systematic literature review (SLR) and meta-analysis according to the Preferred Reporting Items for Systematic Reviews and Meta-Analyses (PRISMA) guidelines; II) review of national health system (NHS) databases and systematic reports; III) physician survey; and IV) an epidemiologic model. ICD 10 D84.1 encoded records from the NHS were used to estimate the number of patients with HAE attended and treated during 2019. A survey was implemented to increase understanding of the clinical profile and treatments used.

**Results:**

A prevalence rate of 0.9/50 000 inhabitants was estimated for 2019. In the same year, an estimated 317 HAE type 1 patients were recorded in the NHS, aged ≥11 years old. The most frequent clinical symptoms were cutaneous edema (67.5%) and abdominal pain (47.9%). A severe episode with laryngeal edema appeared in 27.5% of cases. Acute episodes were mainly moderate to severe (77.0%), with an annual per capita frequency of emergency visits of 7.6 patient-year (range 1–12/patient-year). The main reasons for hospitalization corresponded to laryngeal facial, tongue, and abdominal edemas, representing 73.3% of annual ICD 10 D84.1 reported hospitalizations. The main treatments that patients with HAE received were fresh frozen plasma for acute attacks and danazol for short-term prophylaxis (STP).

**Conclusions:**

Despite efforts to make HAE visible, according to this study, cases recognized and treated in the NHS represent only 16.6% of the estimated prevalence.

## Introduction

Hereditary Angioedema (HAE) is characterized by recurrent, localized, and self-limited angioedema episodes (not accompanied by wheals) involving the skin and/or mucous membranes of the respiratory and gastrointestinal tracts.^1^ Recurrent angioedema can be divided into inherited forms, HAE, and acquired forms, acquired angioedema (AAE).^1^ A genetic C1 inhibitor (C1-INH) deficiency (HAE type 1) or lack of function (HAE type 2) are the causes of the classic and most common types.^1^

HAE is a rare, unpredictable, serious, and potentially fatal genetic disease; 75% are inherited in an autosomal dominant manner, and up to 25% of cases correspond to spontaneous genetic changes (de *novo* mutation).^2^ The genetic background of HAE lies fundamentally in *SERPING1* gene variants, which generates decrease (HAE type 1) or dysfunction without decrease (HAE type 2) of C1-INH. In HAE C1-INH, around 85% cases are HAE type 1 and 15% cases are HAE type 2.^3^

HAE is recognized as a *rare disease* with a worldwide estimated prevalence in the range between 1 case per 50 000 and 1 case per 100 000 inhabitants,^4^ with no identified differences in prevalence due to gender.^5^ Recently published information points to differences in prevalence among ethnic groups (Hispanics, Asians, white and black populations)^6^ and regions (western countries vs Asia Pacific).^7^ Due to its low prevalence and the fact that it shares symptoms with other more frequent diseases, HAE is not usually suspected in the first instance in some areas such as Mexico and other developing countries. This situation leads to misdiagnosis and underdiagnosis contrasting with Europe and developed nations.^8^

Consequently, patients are often treated ineffectively and late, living many years (on average 22), with a significant impact on their quality of life (QoL)^9^ until finally obtaining a correct diagnosis and appropriate treatment. Some of them have even undergone unnecessary surgeries (for example, negative appendectomy) due to lack of disease awareness.^10^

Another potential reason for the underdiagnosis of HAE is the low availability of population-based epidemiological studies worldwide, as well as the existence of few systematized patient registries∗ which explains why the prevalence figures that circulate in literature show great dispersion,^9^ thus making it difficult to accurately estimate the number of patients with HAE.

Acute episodes with recurrent, painful, and very frequently unpredictable edema, especially those that affect respiratory or gastrointestinal mucosa, face, and extremities in their distal segments, are associated with a high degree of morbidity, poor QoL, and even mortality in the case of episodes with laryngeal edema.^3^^,^^9^^,^^12^, ^13^, ^14^

According to the National Center for Technological Excellence in Health, and the Mexican Association of HAE (consulted in June 2022),^15^ in Mexico there are no population-based epidemiological studies, nor an HAE registry of patients.

Finally, given that the current and available data sources of information in Mexico^16^ do not include diagnostic-therapeutic details of HAE patients' care, it is likely that the current estimates of the burden of disease are inaccurate. Therefore, it is also important to compare the shared participation of public and private care for patients with HAE, given the differences in type of patients, budget constraints, and access to both up to date diagnostic methods and specialized care. The aim of this study was to estimate the prevalence in HAE types 1, 2, and nC1-INH, understand the process of diagnosis, medical care, health resources and specific treatments consumption, during acute attacks and prophylactic strategies in Mexico.

## Methodology

A mixed methods approach was conducted in 4 phases: I) Systematic Literature Review (SLR) and meta-analysis; II) review of the national health system (NHS) databases and systematic reports; III) angioedema-specialized physicians (AEP) survey; and IV) an epidemiologic model.

### Systematic literature review and meta-analysis

A systematic literature review (SLR) was carried out to obtain information on HAE: prevalence and frequency of HAE types 1, 2, and nC1-INH. This SLR was conducted according to PRISMA guidelines.^17^ The sources of information used included the following platforms and search engines: electronic library of the National Autonomous University of Mexico (UNAM), National Library of Medicine (NLM, Pubmed), OVID, SCIENCE DIRECT, SciELO and Virtual Health Library (BVS, for its acronym in Spanish). Access to the following data bases was obtained: MEDLINE, EMBASE, LILACS, EMB Reviews, Practice Guidelines, The Cochrane Library, and IMBIOMED.

Literature search was conducted accordingly to find papers with the following inclusion criteria as it is recommended by Moncada-Hernández:^18^ Mexican and international peer reviewed journals indicated in the medical databases; HAE prevalence (number patients/50 000–100 000 inhabitants); patients with an established HAE diagnosis by gender (male, female); patients’ age (0–100 years old); patients by age group (≤10, 11–20, 21–30, 31–40, 41–50, 51–60, and >60 years); HAE type (Type 1, Type 2, or nC1-INH); study classification (systematic reviews, case series, descriptive studies of patient populations, and population-based epidemiological studies). Exclusion criteria: all the HAE papers that do not meet the inclusion criteria (not include HAE by type, gender, age groups, or population explicitly) and conference abstracts.

The key search terms used were all crossed referenced with the terms: HAE or Hereditary Angioedema.

The search strategy included studies published in English or Spanish from January 1999 to April 2022. The articles which met the inclusion criteria were searched for additional references. Exact search algorithms with keywords, Medical Subject Headings terms, entree terms and search strings, and details regarding the SLR are available in Supplement S1. Full-text available studies were included for initial review. Articles were reviewed by 2 authors independently, with discrepancies resolved after joint article review and discussion.^18^

The selected published articles and specialty theses (see details Supplement S2) underwent a meta-analysis with the Comprehensive Meta Analysis® program from the US National Institutes of Health (NIH).

From this data, the prevalence rate of HAE/50 000–100 000, and the frequencies of type 1, type 2, type nC1-INH, female, male, and age groups were obtained, and point values and 95% CI (Confidence Intervals) were calculated.

### Review of Mexican NHS databases and systematic reports

The objective of this phase was to retrieve from the databases and systematic reports of the NHS (public and private) through the records encoded by ICD 10, the number of HAE cases attended during 2019, the distribution of cases (by public and private healthcare, gender, and age group), as well as the number of outpatient consultations, medical emergency consultations services, and hospitalization data events (number, rate, and days of stay >24h).

The following NHS databases and systematic reports for 2019 were reviewed: the statistical yearbooks of the major health institutions in the public sector (97% coverage),^19^, ^20^, ^21^, ^22^ as well as the database of health information in private health units,^23^ the annual report of morbidity^24^ and mortality,^25^ and the report of hospital discharges by specific cause of the general direction of epidemiology of the Ministry of Health (MoH) in Mexico.^26^

For the tracking of cases treated during 2019 ICD code D84.1 was used, which only identifies cases with deficiency C1-INH. Also, it does not differentiate between inherited and acquired cases. The differentiation between hereditary and acquired cases, as well as type 1 or type 2 HAE was estimated with the clinical information obtained through a survey applied to AEP.

Other public database used correspond to the 2020 Population and Housing Census from the National Institute of Geography and Statistics (INEGI).^27^ From here, data on the structure of the Mexican population and the population pyramid for each age group and gender were obtained (the adjusted data for 2019, was also retrieved from INEGI).^28^

In accordance with the above, the estimation of patients cared for in the NHS during 2019 was calculated by the retrieval of records coded with ICD 10 D84.1 from all NHS institutions and the annual report of the private sector for 2019. From the records, the distribution of cases by age group and gender was obtained, as well as the number of outpatient consultations, medical emergency consultations services, and hospitalized patients (number, rate, and days of stay). All the records were added, including the demo-epidemiological and medical care items above mentioned. Thus, the total number of cases registered, attended, and treated in the NHS was obtained. From the total, the share of both the private and public sectors was determined. In the public sector, the share of each one of the institutions was identified.

### Physician survey

To understand the clinical status and treatment of patients with HAE, a survey was designed to collect the data from the AEP (Supplement S3). To develop questions and consolidate the survey, a Delphi Panel^29^ was implemented. For controlling the quality of Delphi methodology, according to Nasa et al,^29^ we committed attention to 9 points: 1) identification of a problem, 2) area of research, 3) panel selection and anonymity, 4) controlled feedback, 5) iterative Delphi rounds, 6) consensus criteria, 7) analysis of consensus, 8) closing criteria, and 9) stability of the results.

We used a convenient sampling strategy to identify AEP to whom the survey was applied. The criteria applied considered physicians from the medical units with the highest number of ICD 10 D84.1 coded records over 2019.^19^, ^20^, ^21^, ^22^, ^23^

The AEP were selected from the hospitals corresponding to the major institutions of the public sector, and for the private sector, tertiary care hospitals in Mexico City. The proportion of AEP was determined according to the share of records obtained from the public and private sectors, respectively. In the case of the public sector, the share among the different institutions was respected.^19^, ^20^, ^21^, ^22^, ^23^

The objective of the survey administered to AEP was to obtain the average clinical profile (signs, symptoms, frequency, and severity of acute attacks), the average treatment used for the acute attack and for short-term prophylaxis (STP) management (aim: prevention of acute attack related to a potential trigger condition, ie, infection, minor or major surgery, etc), as well as validation of the frequency of HAE types, gender distribution, and affected age groups. Also, the proportion of cases of HAE and AAE was estimated with the clinical information obtained through the survey applied to 40 AEP; the relevant data that obtained consensus during the Delphi Panel to filter clinically were: absence of urticaria, recurrent abdominal pain or swelling, lack of response to conventional antiallergic treatment (typically use of histamine Type-2 Receptor Antagonists and glucocorticoids), family history, recurrent angioedema attacks, and average age of symptoms onset (childhood, youth, adult). Other mentioned data ACEI treatment.

Finally, and from the survey also, the differentiation between Types 1 and 2 was made based on laboratory studies carried out (C1-INH levels, C1-INH functional quantification, C1q and C4 levels). The verification rate with the genetic test in relation to tests performed for clinical and laboratory suspicion was also considered. The mean, standard deviation, and 95% CI were calculated from the 40 surveys retrieved from AEP. The reported results correspond to the analysis of the survey responses.

### Epidemiologic model

As mentioned above, there are no reliable epidemiological studies nor population registries of patients with HAE for Mexico.^15^ In this regard, a probabilistic model was built in Microsoft Excel® to obtain specific Mexican calculations.

To estimate the HAE prevalence, a meta-analysis of the information obtained from population studies that considered specific countries and other determined groups was carried out.^30^ The information was only available in aggregate form at the level of total populations by country or specific groups and documented cases - registered by country or population in the same period. The number cases x 100 000 inhabitants were determined for each country or population analyzed.

According to the frequency determined by the panel of AEP, the population estimate for HAE type 1 and type 2 was made. Since the ratio for HAE nC1-INH agreed upon by the panel of AEP was small, it was decided to add it on top of that determined for HAE types 1 and 2. Mean values and 95% CI were calculated.

The model was populated with the specific information obtained (assumed as *proxy* variables) from the following information sources:

- data from the official Mexican population (2019)^27^^,^^28^
- the results of the meta-analysis (mean values and 95% CI) of the literature reviewed
- epidemiology data extracted from the electronic pages (statistical reports and yearbooks) of Orphanet^31^ and the Mexican Federation of Rare Diseases^32^
- distribution by gender and age group of NHS ICD code D84.1 records retrieved for 2019^19^, ^20^, ^21^, ^22^^,^^26^

The schematic mechanics of the modeling process is shown in Supplement S4, in brief: to estimate prevalence for Mexico, a meta-analysis obtained from population studies that considered specific countries and other determined groups was carried out. The mean value and scenario with a triangular distribution was determined from the extreme maximum-minimum values available for the populations. With these, health modeling was carried out using a first-order Monte Carlo simulation with 10 000 iterations. Mean values and 95% confidence interval (CI) were estimated as rates. From this, we calculated the number of total HAE cases (C1-INH deficiency) for the total Mexican population in 2019.

## Results

**Fig. 1:**
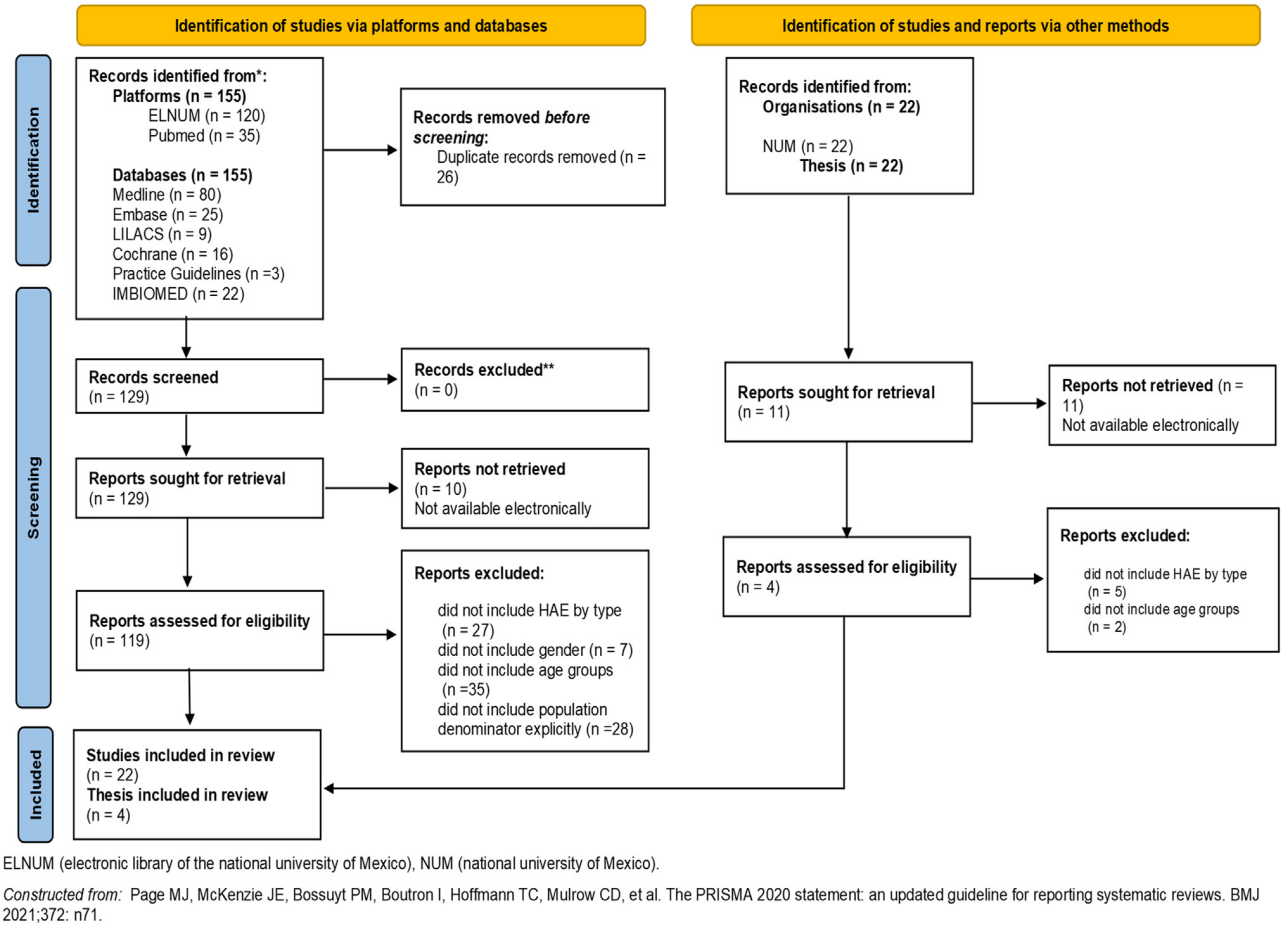
PRISMA diagram of the systematic literature review (SLR)

SLR PRISMA flow diagram^17^ is shown in Fig. 1. From the meta-analysis of the SLR, the Mexican HAE estimated prevalence rate was 0.805/50 000 inhabitants (CI 95% 0.805–0.808). Using this estimated prevalence and considering Mexico's gender distribution reported in 2019,^28^ women with HAE represents 54.1% (CI 95% 48.0%–76.7%), and 45.9% (CI 95% 23.3%–52.0%) for men. For HAE type, data obtained by the AEP survey adjusted by estimated prevalence showed that 84.2% (CI 95% 83.7%–84.8%) corresponded to type 1, 14.9% (CI 95% 14.8%–15.0%) were type 2, and 0.9% (CI 95% 0.9%–0.9%) to nC1-INH. Absolute cases calculated with this model showed a prevalence of 2024 total cases for 2019 (0.805/50 000 inhabitants). From these and according to the output analysis of the survey administered to AEP, it was considered an average of 12.3 acute attacks/year (range 4.0–27.1), the average severity of acute attacks, corresponded to mild in 23.0%; moderate in 50.0%, and severe in 27.0% (Table 1).

**Table 1:** Hereditary Angioedema: Estimated prevalence. Cases by gender. Cases by Type. Acute Attack by average severity. Acute Attack by annual average per capita. Specific diagnosis rate (ICD 10: D84.1) in Mexico, 2019

Absolute cases = 2024	
Prevalance 0.805/50,000 inhabitants	
**Variable**	**n (%)/CI 95%**
**Gender**	
Man	929 (45.9)/23.3–52.0
Women	1095 (54.1)/48.0–76.7
**HAE type**	
Type 1	1704 (84.2)/83.7–84.8
Type 2	302 (14.9)/14.8–15.0
Type nC1-INH	18 (0.9)/0.9–0.9
**Acute Attack severity**	
Mild	6256 (23)
Moderate	13942 (50)
Severe	7555 (27)
**Attack recurrency**	12.3 (4.0–27.1)
**Actual/Estimated HAE prevalence**	376/2024 (18.57)

Regarding HAE cases, reported in the NHS (determined from the review of the NHS databases and systematic reports, specifically through ICD 10 D84.1 coded records [January 2019–December 2019]), 376 were identified as C1-INH deficiency (public and private) cases. From this and based on what was obtained from the survey applied to AEP, it was estimated that 352 patients were HAE (93.6% [95% CI 91.2%–95.2%]), and 24 (6.4% [95% CI 5.9%–9.3%]) cases AAE. From the 352 HAE cases, 317 were estimated as HAE Type 1 (90.1% [95% CI 84.3%–91.1%]), and 35 cases were considered HAE Type 2 (9.9% [95% CI 9.2%–10.4%]). On the other hand, patients with nC1-INH were not considered for the present analysis since there is no specific or suggestive ICD-10 code for them. The AAE cases were not considered for further analysis either.

Of the 352 HAE cases, seen and treated during 2019 within the NHS, the distribution by age groups of the patients was as follows: ≤10 years 32.0% (113); 11–20 years 19.5% (69); 21–30 years 10.5% (37); 31–40 years 11.2% (39); 41–50 years 6.8% (24); 51–60 years 9.1% (32) and >60 years 10.9% (38). The average number of consultations was 7/patient-year (Table 2).

**Table 2:** Hereditary Angioedema (ICD 10 D84.1 defects in the complement system): Outpatient care, inpatient care, hospital cases and main causes of hospitalization. Distribution according to age groups and Institutions in Mexico, 2019

**Outpatient care (Ambulatory)**									
Age	≤10 years	11–20 years	21–30 years	31–40 years		41–50 years	51–60 years	>60 years	Total
Patients seen	113	69	37	39		24	32	38	352
Distribution	32.0%	19.5%	10.5%	11.2%		6.8%	9.1%	10.9%	100.0%
**Average consultations = 7/year per capita**									
**National health system share**									
**Public Sector** (77.7%)									
**IMSS** (43.8%)									
**SSA** (41.9%)									
**ISSSTE** (10.8%)									
**Others**: Armed Forces (SEDENA, SEMAR), and Mexican Oil Company (PEMEX) (3.5%)									
**Private Sector** (22.3%)									
**Inpatients (hospitalized)**									
Cases	25	18	38	25		11	20	17	154
Distribution	16.2%	12 .0%	24.8%	16.2%		6.8%	12.8%	11.1%	100.0%
**Average hospitalization = 1.61/year per capita**									

**Institution**		**Main Reasons for Emergency Service Attention (location)**		**Main Reasons for Hospitalization (location)**	
**Public Sector**	81.4%	**Subcutaneous**	81.0%	**Abdomen severe edema (simulating acute abdomen)**	8.9%
**IMSS**	62.7%	**Abdominal**	70.2%	**Facial edema**	26.7%
**SSA**	26.7%	**Laryngeal edema**	28.4%	**Laryngeal edema**	24.4%
**ISSSTE**	8.0%	**Mixed**	29.9%	**Tongue edema**	13.3%
**Others**	2.6%			**Severe upper limb edema**	1.2%
**Private Sector**	18.6%			**Severe lower limb edema**	1.0%
				**Edema in >2 locations including face and neck**	20.1%
				**Other**	4.4%

IMSS, Instituto Mexicano del Seguro Social; SSA, Secretaría de Salud; ISSSTE, Instituto de Seguridad y Servicios Sociales de los Trabajadores del Estado.

Regarding hospitalized cases (154), the distribution by age groups of admitted patients was: ≤10 years 16.2% (25); 11–20 years 12.0% (18); 21–30 years 24.8% (38); 31–40 years 16.2% (25); 41–50 years 6.8% (11); 51–60 years 12.8% (20) and >60 years 11.1% (17), with 248 hospital discharges in the year, representing a hospitalization rate of 43.8% in 2019, and an average of 1.61 hospitalizations/patient-year (Table 2).

From the NHS databases and systematic reports consulted relative to the medical care site of these patients, 77.6% of the cases (273) were seen in the public sector: 43.8% (120) in the IMSS (Mexican Social Security Institute), 41.9% (114) in the SSA (National MoH), 10.8% (29) in the ISSSTE (Institute of Security and Social Services for Federal Government Workers) and 3.5% (10) in other institutions. In the private sector 79 patients were cared for 22.3% (Table 2).

The main causes of hospitalization corresponded to facial, tongue, larynx, face, and neck edema (68.8%), and severe edema in the abdomen simulating acute abdomen (8.9%), as well as edema in 2 or more body segments (20%), and important distal limb edema (2.2%). Regarding hospitalization within the NHS, 81.4% of the cases (125) were hospitalized in the public sector, while 18.6% (29) were admitted in private sector (Table 2). The distribution of cases exemplifies the complexity of the health system and potential associated challenges for patients since the private and public sectors are not interconnected. Moreover, within the public sector there is no processes to reference patients, making challenging to have opportune diagnosis, treatment, and follow-up.

**Fig. 2:**
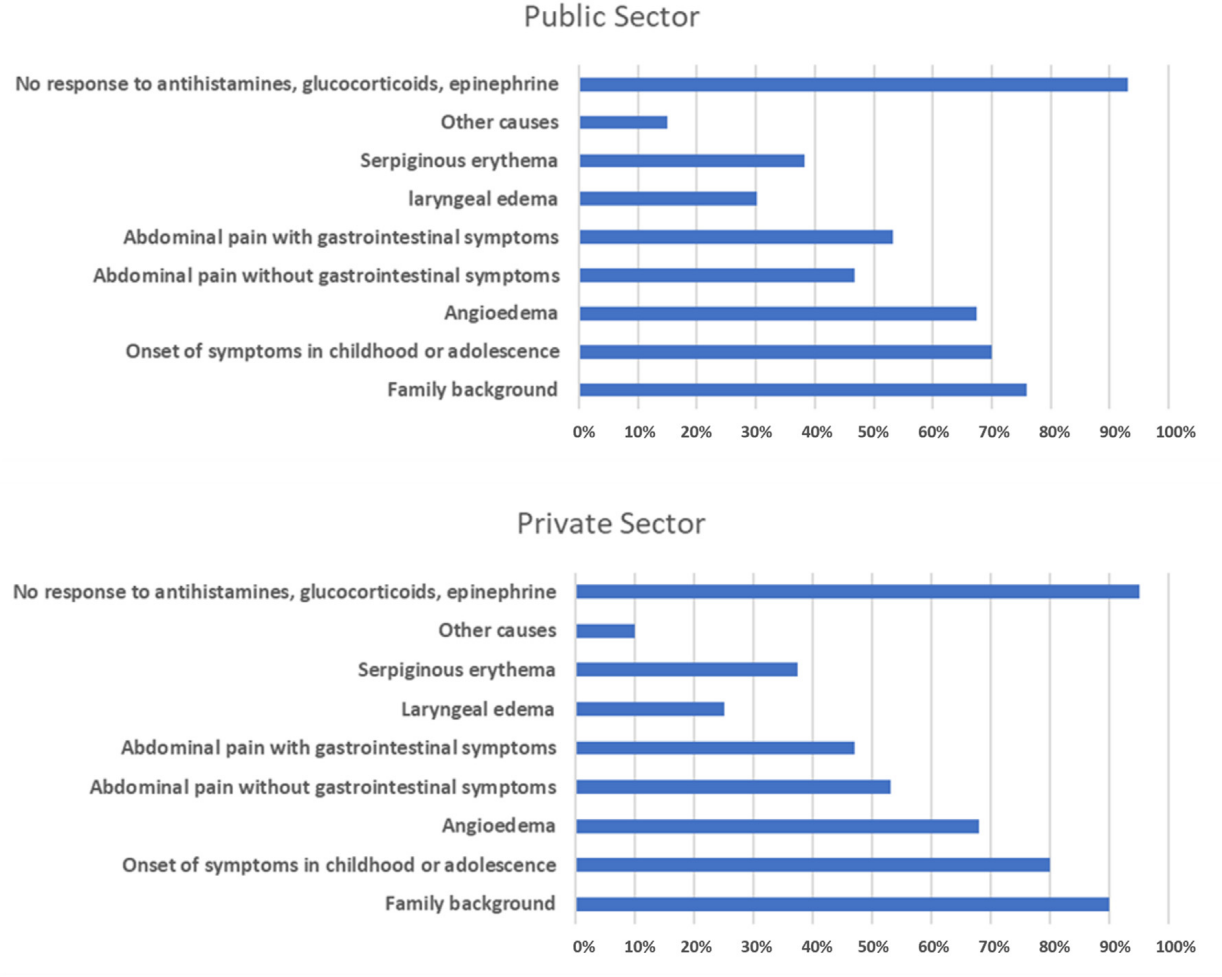
**Hereditary Angioedema (**ICD 10 D84.1 defects in the complement system)**: Main clinical data. Mexico, 2019**

According to the survey applied to AEP, emergency care in medical units, was granted to 33% of patients, being 67.3% men and 32.7% women. The average number of visits was 7.6/patient-year (range 1–12/patient-year). They represented 61.2% of all acute attack care. The rest of the acute attacks of minor severity, were attended at home (13.5%), and in outpatient consultation (25.3%) (Table 2). Regarding findings suggesting HAE, AEP reported the presence of repeated angioedema, with approximately 40–50% of cases involving abdominal pain, lack of response to antihistamines and corticosteroids in 90–95%, family history (76% public sector, 90% private sector), and beginning of symptoms in childhood or adolescence (70% public sector, 80% private sector). From a clinical perspective, the main prodrome were: abdominal pain with associated gastrointestinal symptoms (diarrhea and nausea-vomiting) 47.9% and serpiginous erythema 38.0%, continuing during the clinical manifestation. The main manifestations were in terms of frequency: skin edema 67.5%, abdominal pain without gastrointestinal symptoms 42.1%, laryngeal edema 27.5%, and other symptoms 2% (Fig. 2). The key diagnostic studies carried out in these patients corresponded to the determination of C1-INH levels, C1-INH functional quantification, C1q, C3, and C4 levels (36% in public sector and 50% in private sector). In cases clinically suspected to be acquired, anti-C1INH autoantibodies were determined (15% in both sectors).

Genetic study was carried out in a small group of cases (10%), being pathogenic variants in the *SERPING1* gene the finding in 74% of the patients with genetic test. Regarding acute attack frequency of 12.3 per capita per year (range 4.0–27.1) is mentioned by the AEP (Table 1), being more frequent after adolescence with around 60% of the events in those over 17 years of age (Data not shown).

According to the survey responses analysis to the therapeutic approach, fresh frozen plasma was the most used medical treatment for the acute attack in both public and private sectors, followed by icatibant and C1-INH concentrate. On the other hand, danazol was the most used treatment for STP (Table 3).

**Table 3:** **Hereditary Angioedema (**ICD 10 D84.1 defects in the complement system)**: Specific Treatment of Acute Attack and STP. Mexico, 2019**

Clinical Situation	%	
	Public sector	Private sector
**Acute Attack**		
Icatibant	31.3%	10.0%
Fresh frozen plasma	44.0%	50.0%
C1-INH concentrate	10.9%	10.0%
**Others**	13.8%	30.0%
**STP**	30.2%	25.0%
Danazol	65.0%	70.0%
Epsilon amino caproic acid	15.0%	10.0%
Others	10.0%	5.0%

## Discussion

Mexican nationwide HAE prevalence for 2019 was estimated to be of 2024 total cases, corresponding to a rate of 0.805/50 000 inhabitants (CI 95% 0.805–0.808), with an estimated of 376 patients identified as C1-INH deficiency.

From this data, it was estimated that 352 patients were HAE (93.6% [95% CI 91.2%–95.2%]), and 24 (6.4% [95% CI 5.9%–9.3%]) were AAE cases. From the 352 HAE cases, 317 were estimated as HAE Type 1 (90.1% [95% CI 84.3%–91.1%]), and 35 HAE Type 2 (9.9% [95% CI 9.2%–10.4%]).

The results obtained with this methodology show consistency with previously published data. Banerji et al^13^ (2020) reported 78.4% HAE type 1 in the United States US) population. In the Austrian registry^33^ 80.2% and in Brazilian cohort^34^ 76.1% were HAE type 1. Comparatively, in our study corresponds to 90.1% (n = 317) HAE Type 1 in estimated total population prevalence. Regarding the presence of a family history, Banerji et al^13^ found it in 78.4% of their population. In this study it was estimated in ≥75.0%, in the Austrian registry^33^ 83.7%, and in Brazilian cohort 86.9%.^34^ Regarding the onset of symptoms in these 4 populations it occurred mainly in childhood-adolescence.^13^^,^^33^^,^^34^

Considering the severity of the attacks, in our results from the Mexican and Brazilian populations, around 75% of the events were moderate to severe, with the difference that in Mexico 50% of the cases corresponded to moderate, while in Brazil just over 50% were serious.^34^ In general terms, a similar pattern is observed in various countries, with the sum of moderate plus severe attacks dominating. That implicitly shows that the problem is not close to being controlled. The enormous burden of disease showed by Banerji et al^13^ for the US population allows us to see this clearly: Anxiety and depression were frequently identified, affecting 35.3% and 20.9% of patients, respectively.^13^ Considering these results and the psychological impact on patients, future studies describing this type of implication should be addressed.

Regarding the number of attacks, Banerji et al^13^ found for a period of 6 months prior to the application of a specific survey, an average of 11.1 events per capita in the US population, similar to our results (12.3 average events/patient-year).

Also, Banerji et al found that most patients (78.7%) reported an attack in the last month and that 41.8% suffered an attack within the previous week of the survey's application.^13^

The high number of attacks in frequency, together with their duration for certain patients (33.9% suffered attacks lasting ≥2 days), should be further evaluated since it correlates with negative effects on QoL, as well as on the mental sphere.^12^ It certainly represents a clear area of opportunity to incorporate human development dimensions (QoL, days of productivity lost, mental distress, among others) to HAE research.

The NHS share for public institutions (70.7%, Table 2) represents a big challenge in Mexico because the gross domestic product assigned to health attention is below the recommended by the OCDE. (5.4 vs 8.8, respectively 2021).^35^

The main strength of the study was identified in the methodological approach with the use of mixed methods (carried out in 4 phases: a systematic review of the literature and meta-analysis; review of the NHS databases and systematic reports; Delphi panel and survey; and an epidemiologic model). This allowed the prevalence of HAE in Mexico to be estimated for the total population.^27^^,^^28^ Likewise, by obtaining and adding the records coded by D84.1 of the ICD 10 of all the different databases of the most relevant public institutions and the private sector of the NHS (1/1/2019–31/12/2019),^19^, ^20^, ^21^, ^22^, ^23^ it was possible to identify the number of cases seen and treated in the NHS during 2019, as well as the corresponding distribution by gender and age group, both for outpatients and inpatients. The clinical profile and therapeutic behavior obtained from the *ad hoc* survey results applied to 40 Mexican AEP gave specific details on HAE diagnosis and treatment as real-world experience.

It is worthy to mention that our study has intrinsic limitations regarding the use of administrative databases, such as the lack of clinical, socioeconomic, and QoL variables, a fact that imposes the need for using other sources to correct the information gap, thus implicating the additional risk of introducing bias in the interpretation of information coming from different sources. Likewise, it is important to keep in mind that the registry encoded by ICD 10 D84.1 identifies only cases with C1-INH deficiency but does not differentiate between Type 1 or 2, and of inherited and acquired cases. In relation to the above, in the present investigation, we estimated HAE cases through data from the survey administered to AEP. According to this, the total number of records considered HAE was 352 (96.3%), 317 were estimated as HAE Type 1 (90.1% [95% CI 84.3%–91.1%]), and 35 cases were considered HAE Type 2 (9.9% [95% CI 9.2%–10.4%]). In recent international literature, similar criteria are used to filter or differentiate between hereditary and acquired cases.^36^^,^^37^

Summarizing, it is known that EMR (electronic medical record) databases for investigating HAE in retrospective models can be challenging due to the lack of specific diagnostic codes for this condition, combined with the frequency of delayed diagnosis and/or misdiagnosis.^38^ In this regard, there are structured EMRs such as ICD 10 D84.1, and unstructured ones such as medical notes.^38^ Given that HAE is a rare disease, being able to count on both categories of information, elements that provide more specific data for a more accurate diagnosis that make it possible to estimate hereditary cases from early stages due to C1-INH deficiency qualitatively or quantitatively, or with normal C1 esterase inhibitor, will undoubtedly be key elements for a better understanding and management of this clinical condition. In addition to testimonial information from AEP could and/or considered to add value.^38^

Despite the recognized usefulness of structured EMRs, there are recent reports that show underrepresentation in the number of patients.^38^ Likewise, the value of adding more sources of information has also been documented.^38^ In this study, the addition of the survey supports the results. Above all, relying just in ICD 10 D84.1 could be a risky situation, since this code is just related to C1-INH deficiency, thus, leaving out even the chance of a proxy for estimate HAE nC1-INH, the estimation with the present methodology accounts just for 90%–95% of all HAE cases.

On the other hand, administrative databases have many advantages in an environment where health data are scarce (such as Mexico) as a valuable, standardized, and systematic resource. Although our methodology to estimate prevalence and frequency *proxies* is not a substitute for other designs (population-based epidemiological studies, an accurate and systematic National HAE registry), it is less expensive (economically and in run time) and more convenient than prospective follow-up studies due to the difficult of enrolling significant amounts of patients in a reasonable period. Future designs in settings where electronic comprehensive health records are available, could include detailed information on treatment and clinical manifestations per capita.

These results are considered to provide an interesting and innovative approach in the Mexican level to estimate and understand the calculated burden of HAE in Mexico, as well as an additional step in understanding the diagnose and treatment profile of the disease locally.

The current ICD 10 version for 2019 considered the ICD 10 code D84.1 to describe cases with C1-INH deficiency.^39^ Although there are other complement system deficiencies documented and considered by this ICD 10 code, as shown in the specialized literature^40^ other different to C1 inhibitor deficiency, account for less than 15% (and individually being very rare), when using the mapping for the determination of the applicable DRG, the meaning most frequently found when using ICD 10 D84.1, substantially, corresponds to its meaning as C1-INH deficiency. Therefore, and according to the panel of AEP, as well as some regional clinical practice guidelines and the health information system of the Mexican MoH, this code is considered an adequate proxy to identify and follow up on cases of C1-INH deficiency, whether acquired or hereditary and of these Type 1 or Type 2. This coding does not allow to directly differentiate between HAE Type 1 and Type 2 cases, as well as between hereditary and acquired cases of angioedema, for this, other sources of information and health modeling must be used.

In support of consideration and use as a proxy for C1-INH deficiency analysis, it is known that this ICD 10 code, in practice (diagnostic coding) is related to the following ORPHANET codes: ORPHA 528663 (AE acquired); ORPHA 528623 (hereditary AE), ORPHA 100050 (HAE Type 1), ORPHA 100051 (HAE Type 2), and with MS-DRG 40.0.

With the advent of ICD 11, it is now possible to differentiate directly from the coding, between cases of AE specifically (no longer including the code to other complement deficiencies). The specific ICD 11 codes are ICD 11 4A 00.14 (HAE) and ICD 11 4A 00.15 (AAE). Despite this, in order to discriminate between the different types of HAE, it will still be necessary to use complementary methods such as medical notes and surveys with treating physicians. This implies knowing firsthand, at least the number of cases of HAE and AAE.

## Conclusions

The present study represents the first publication that provides an estimate of the number of patients with HAE (focusing on Type 1 which represents more than 90% of the cases), by gender and age in Mexico based largely on local data sources. It also includes data on epidemiology, diagnosis, presentation, attack characteristics, healthcare processes, and treatment patterns to increase the understanding of HAE in the Mexican population. It is important to note that according to this study, the recognized and treated cases of HAE in the NHS in a full year (2019) represent only 16.6% of the estimated prevalence for 2019.

## Abbreviations

3-OST-6, 3-O-sulfotransferase 6; ACMG–AMP, American College of Medical Genetics and Genomics–Association for Molecular Pathology; AAE, Acquired angioedema; ACEI, Angiotensin Converting Enzyme Inhibitors; AEP, Angioedema Experts physicians; BVS, Virtual Health Library; C1-INH, C1-esterase inhibitor; C1-INH-HAE, HAE due to C1 inhibitor deficiency; CI, Confidence Intervals; DNA, Deoxyribonucleic Acid; EMR, Electronic Medical Record; FXII-HAE, HAE due to variants in the factor 12 gene; HAE, Hereditary angioedema; HAE-ANGPT1, HAE due to variants in angiopoietin 1; HAE-KNG1, HAE due to variants in kininogen-1; HAE-MYOF, HAE due to a gain-of-function variant in myoferlin genes; HAE-PLG, HAE due to variants in plasminogen; HRQoL, Health-Related Quality of Life; HS, Heparan sulfate; ICD 10 D84.1, International coding of diseases in its 10th revision, code D84.1; IMSS, Mexican Social Security Institute; INEGI, National Institute of Geography and Statistics; ISSSTE, Institute of Security and Social Services for Federal Government Workers; MeSH, Medical Subject Headings; MYOF, Myoferlin; MoH, Ministry of Health; nC1-INH, normal C1-INH; NHS, National Health System; PEMEX, Mexican oil; PRISMA, Preferred Reporting Items for Systematic Reviews and Meta-Analyses; QoL, Quality of Life; SciELO, Scientific Electronic Library Online; STP, Short-Term Prophylaxis; SEDENA, National Defense Secretariat; SEMAR, Marine Secretary; SERPING1, Serpin family G member 1; SLR, Systematic literature review; SSA, Health Ministry of Mexico; U-HAE, HAE cases in which the underlying defect is still unknown.

## Funding

This study was sponsored by Takeda Mexico, S.A. de C.V. Medical writing support, under the guidance of the authors, was provided by Alfonso Rosado, MD, MSc, PhD and Links and Links, and was funded by Takeda Mexico, S.A. de C.V.

## Availability of data and materials

Data and materials available within the article or its supplementary materials. A) systematic literature review, search terms and strings available on supplemental material 1; B) meta-analysis using Comprehensive Meta Analysis® version 3.3070; C) public and available databases analysis, all mentioned in the manuscript; D) physician survey, material for data collection available on supplemental material 3. Considering that there was not collected information from individual patients, an Ethics Committee approval was not required according to national regulation.

## Authors Contributions and publication consent

María Eugenia Vargas. Manuscript writing, critical revision, final approval to submit. She provided consent for publication
Sandra Nieto. Manuscript writing, critical revision, final approval to submit. She provided consent for publication
Ileana Madrigal. Manuscript writing, critical revision, final approval to submit. She provided consent for publication
Francisco Contreras. Manuscript writing, critical revision, final approval to submit. He provided consent for publication

## Acknowledgements

In memory of Dr. Nora Hilda Segura Méndez for her invaluable contribution. Rest in peace.

## Declaration of competing interest

María Eugenia Vargas has served on the advisory board of Takeda Mexico, CSL Behring, GSK and Liomont advisory. She has also been a speaker with Takeda Mexico, CSL Behring, Astra Zeneca. She has received academic support (scholarships) from Takeda Mexico, CSL Behring, Astra Zeneca and Boehringer. Sandra Nieto has been a speaker for Takeda Mexico, CSL-Behring, Pint-Pharma. She has been a consultant for Takeda Mexico. She has participated in research protocols for Takeda (Principal Investigator). Ileana Madrigal has been a speaker for Takeda Mexico. Francisco Contreras has been a paid speaker for Takeda and CSL Behring. He has participated in research protocols for Takeda.

## Footnotes

Appendix A Supplementary data to this article can be found online at https://doi.org/10.1016/j.waojou.2023.100812.

## Footnotes

∗ AN: After reviewing the specialized literature for the writing of this paper, it is clear to the authors that there is a small number of publications or specialized communications about case records or population-based epidemiological studies on this disease.^4^^,^^8^^,^^11^
