# Supplementary material for: Real-world experience of hereditary angioedema (HAE) in Mexico: A mixed-methods approach to describe epidemiology, diagnosis, and treatment patterns
Source: World Allergy Organ J. 2023 Sep 13;16(9):100812. doi: 10.1016/j.waojou.2023.100812 (PMC10506135; doi:10.1016/j.waojou.2023.100812)
Supplement: Multimedia component 1 [file mmc1.docx]

**Supplement 1**

Specific search terms and strings used in the Systematic Literature Review strategy. SLR Summary.

**ELECTRONIC LIBRARY OF THE NATIONAL AUTONOMOUS UNIVERSITY OF MEXICO**

<https://www.bidi.unam.mx/index.php/herramientas-de-busqueda/busqueda-en-el-sitio>

advanced search

- Date: 01/01/1999 to 04/30/2022

hereditary AND angioedema AND prevalence *

hereditary AND angioedema AND prevalence AND spanish*

hereditary AND angioedema AND Mesh AND prevalence*

hereditary AND angioedema AND Mesh AND prevalence AND spanish*

hereditary AND angioedema AND prevalence AND women*

hereditary AND angioedema AND prevalence AND women AND spanish*

hereditary AND angioedema AND prevalence AND female*

hereditary AND angioedema AND prevalence AND female AND spanish*

hereditary AND angioedema AND prevalence AND man*

hereditary AND angioedema AND prevalence AND man AND spanish*

hereditary AND angioedema AND prevalence AND male*

hereditary AND angioedema AND prevalence AND male AND spanish*

hereditary AND angioedema AND prevalence AND age group*

hereditary AND angioedema AND prevalence AND age group AND spanish*

hereditary AND angioedema AND prevalence AND age AND distribution*

hereditary AND angioedema AND prevalence AND age AND distribution AND spanish*

hereditary AND angioedema AND prevalence AND age AND specific AND distribution*

hereditary AND angioedema AND prevalence AND age AND specific AND distribution AND spanish*

hereditary AND angioedema AND prevalence AND age AND specific AND distribution AND type*

hereditary AND angioedema AND prevalence AND age AND specific AND distribution AND type AND spanish*

hereditary AND angioedema AND prevalence AND age AND specific AND distribution AND type *

hereditary AND angioedema AND prevalence AND age AND specific AND distribution AND type AND spanish*

hereditary AND angioedema AND prevalence AND age AND specific AND distribution AND C1-INH-HAE AND type 1 AND type 2 AND nC1-INH *

hereditary AND angioedema AND prevalence AND age AND specific AND distribution AND C1-INH-HAE AND type 1 AND type 2 AND nC1-INH AND spanish*

hereditary AND angioedema AND prevalence AND frequency AND specific AND distribution AND C1-INH-HAE AND type 1 AND type 2 AND nC1-INH *

hereditary AND angioedema AND prevalence AND frequency AND specific AND distribution AND C1-INH-HAE AND type 1 AND type 2 AND nC1-INH AND spanish *

n = 120

Medline = 62

Embase = 17

LILACS = 9

Cochrane = 5

Practice Guidelines = 3

Imbiomed = 22

Records = 22

Thesis (postgraduate_allergy and clinical immunology (adults/pediatrics) = 22

advanced search

- Date: 01/01/2015 to 04/30/2022

hereditary AND angioedema AND prevalence *

hereditary AND angioedema AND prevalence AND spanish*

hereditary AND angioedema AND Mesh AND prevalence*

hereditary AND angioedema AND Mesh AND prevalence AND spanish*

hereditary AND angioedema AND prevalence AND women*

hereditary AND angioedema AND prevalence AND women AND spanish*

hereditary AND angioedema AND prevalence AND female*

hereditary AND angioedema AND prevalence AND female AND spanish*

hereditary AND angioedema AND prevalence AND man*

hereditary AND angioedema AND prevalence AND man AND spanish*

hereditary AND angioedema AND prevalence AND male*

hereditary AND angioedema AND prevalence AND male AND spanish*

hereditary AND angioedema AND prevalence AND age group*

hereditary AND angioedema AND prevalence AND age group AND spanish*

hereditary AND angioedema AND prevalence AND age AND distribution*

hereditary AND angioedema AND prevalence AND age AND distribution AND spanish*

hereditary AND angioedema AND prevalence AND age AND specific AND distribution*

hereditary AND angioedema AND prevalence AND age AND specific AND distribution AND spanish*

hereditary AND angioedema AND prevalence AND age AND specific AND distribution AND type*

hereditary AND angioedema AND prevalence AND age AND specific AND distribution AND type AND spanish*

hereditary AND angioedema AND prevalence AND age AND specific AND distribution AND type *

hereditary AND angioedema AND prevalence AND age AND specific AND distribution AND type AND spanish*

hereditary AND angioedema AND prevalence AND age AND specific AND distribution AND C1-INH-HAE AND type 1 AND type 2 AND nC1-INH *

hereditary AND angioedema AND prevalence AND age AND specific AND distribution AND C1-INH-HAE AND type 1 AND type 2 AND nC1-INH AND spanish*

hereditary AND angioedema AND prevalence AND frequency AND specific AND distribution AND C1-INH-HAE AND type 1 AND type 2 AND nC1-INH *

hereditary AND angioedema AND prevalence AND frequency AND specific AND distribution AND C1-INH-HAE AND type 1 AND type 2 AND nC1-INH AND spanish *

**NATIONAL LIBRARY OF MEDICINE**

<https://pubmed.ncbi.nlm.nih.gov/>

**advanced search**

- Date: 01/01/1999 to 04/30/2022

**PubMed Database search**

((((angioedema, hereditary[MeSH Terms]) OR ("hereditary angioedema"[Title/Abstract])) OR ("hae"[Title/Abstract])) OR ("hereditary angioedemas"[Title/Abstract])) AND (((((((((("type 1/type 2/ nC1-INH "[MeSH Terms]) OR ("hae by type "[MeSH Terms])) OR ("hae distribution by age"[MeSH Terms])) OR ("hae distribution by age group"[MeSH Terms])) AND ("hae distribution by gender/male/female"[MeSH Terms])) OR (“haetype1”[Title/Abstract])) OR (“haetype2” [Title/Abstract])) OR (“hae nC1-INH” [Title/Abstract]))

OR

((((angioedemas, hereditary, prevalence [MeSH Terms]) OR ("hereditary angioedema prevalence"[Title/Abstract])) OR ("hae prevalence"[Title/Abstract])) OR ("hereditary angioedemas prevalence"[Title/Abstract])) AND ((((“hae prevalence by age group”[Title/Abstract]) OR (“hae prevalence by gender”[Title/Abstract] [Title/Abstract])) OR (“hae cases/study” [MeSH Terms])) OR (“hae population based/study” [MeSH Terms])) OR (“hae population based/epidemiol*study” [MeSH Terms]))

n = 35

Medline = 18

Embase = 8

Cochrane = 9

Summarizing: Database search identified 155 articles for review and 22 additional records were identified.

After removal of duplicates and screening based on title and abstracts, 119 full text articles and 4 theses were assessed for eligibility. After inclusion and exclusion criteria were applied a total of 26 records were included in the final review (22 studies and 4 theses of specialty in allergy and clinical immunology). PRISMA flow diagram is available for reference in Figure 1 of the main text.
