# Supplementary figures and images for: Real-world experience of hereditary angioedema (HAE) in Mexico: A mixed-methods approach to describe epidemiology, diagnosis, and treatment patterns
Source: World Allergy Organ J. 2023 Sep 13;16(9):100812. doi: 10.1016/j.waojou.2023.100812 (PMC10506135; doi:10.1016/j.waojou.2023.100812)

**Supplement 2**

Overview of the 22 papers and 4 theses. Prevalence estimation.


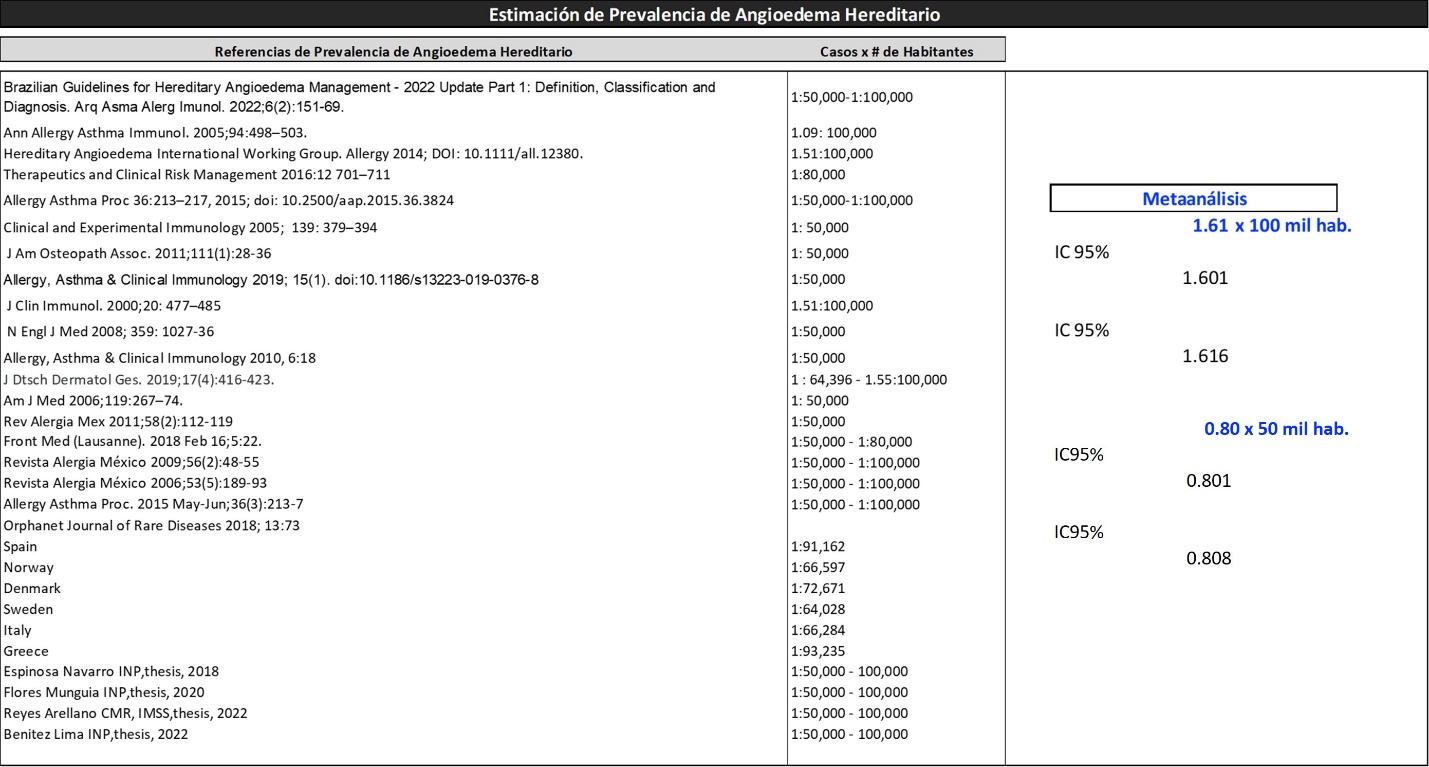

Supplement: Multimedia component 2 [file mmc2.docx]
