# Supplementary material for: Real-world experience of hereditary angioedema (HAE) in Mexico: A mixed-methods approach to describe epidemiology, diagnosis, and treatment patterns
Source: World Allergy Organ J. 2023 Sep 13;16(9):100812. doi: 10.1016/j.waojou.2023.100812 (PMC10506135; doi:10.1016/j.waojou.2023.100812)
Supplement: Multimedia component 3 [file mmc3.docx]

**Supplement 3**

**Survey applied to local treating physicians (Experts) HEREDITARY ANGIOEDEMA (HAE) in the National Health System (Public Sector and Private Sector) 2019.**

| **Variables Patients and Diagnosis / Hereditary Angioedema (HAE)** | |
| --- | --- |
| 1. Of your medical consultation, how many consultations do you grant for **ALL CAUSES**per month?     #_____________consultations | ***Mark with an X":***  a)    <30 per month______  b)    30 to 40 a month ______  c)    40 to 50 per month ______   1. 50 to 60 a month ______ 2. 60 to 70 a month ______ 3. F)      70 to 80 per month______ 4. g)    80 to 90 per month______ 5. g)    90 to 100 a month ______ 6. > 100 per month ______ |
| 1. Of all your **patients**, how many patients do you manage per month with **Hereditary Angioedema ( HAE )?** | ***Mark with an X":***   1. < 5 per month \|__\| 2. 5 to 10 per month \|__\| 3. 10 to 20 per month \|__\| 4. 20 or more \|__\|   ***#***______ patient |
| 1. Of all your patients with **Hereditary Angioedema (HAE)**, on average, how many consultations do you provide per month to patients…   *****NOTE: At the end of the questionnaire, it is defined that it is a suspect, a probable and with an accurate diagnosis*** | ***Write # of patients***  ***#_____________________ suspects***  ***#_____________________ likely***  ***#_____________________ with confirmed diagnosis*** |
| 1. On average, how many **NEW cases do you see**per month with **Hereditary Angioedema (HAE)**? | ***Write # of patients***  ***#_____________________ suspects***  ***#_____________________ likely***  ***#_____________________ with confirmed diagnosis*** |
| 1. Of all your patients with **Hereditary Angioedema (HAE)**, what percentage are: | ***Write the %, it should add up to 100%***   1. First time_________________% 2. Subsequent_______________% |
| 1. Of all your patients with **Hereditary Angioedema (HAE)**, what percentage corresponds to...? | ***Write the %, it should add up to 100%***   1. Men______% 2. Women _______% |
| 1. Of all your patients with **Hereditary Angioedema (HAE)**, how are they distributed by age group (%)? | ***Write the %, it should add up to 100%***   1. < 20 years old________% 2. 20 to 30 years old________% 3. 30 to 49 years old ________% 4. 50 to 59 years old ________% 5. 60 to 69 years old ______% 6. 70 to 79 years______% 7. > 80 years old________% |
| 1. Of all your patients, what % of patients come to the consultation with a previously established diagnosis of **HAE**? | %__________ |
| 1. Of all your diagnosed patients, what is the % of patients with **HAE …**? | ***Write the %, it should add up to 100%***   1. Treated_______% 2. Untreated_______%   ***(refers to disease-specific treatment)*** |
| 1. How many consultations per year does an average **HAE patient require**?   **(inquire minimum and maximum extremums)** | #__________ |
| 1. Of all your patients with **HAE**, what percentage are attributed to the following causes? | ***Write the %, it should add up to 100%***     1. Recurrent angioneurotic edema with nausea and vomiting ___________(%) 2. Edema of limbs (lower and upper __________(%) 3. Edema in the pelvic region in women _____(%) 4. Edema in the pelvic region in men_____(%) 5. Laryngeal edema___________(%) 6. Defects in the complement system___________(%)  - which one specify - _________________ - _________________ - _________________ |
| 1. What percentage of all your patients with **HAE**present edema in the following body areas? | ***Write the %, it should add up to 100%***     1. Skin (Face and extremities) ___________(%) 2. Abdominal, with abdominal pain ___________(%) 3. Abdominal, without abdominal pain ___________(%) 4. Upper airway___________(%) 5. Others___________(%)  - Which ones, specify (%) - _________________ - _________________ - _________________ |
| 1. What percentage of the total of your patients with **HAE**, presents the following degrees of severity in the crises? | ***Write the %, it should add up to 100%***     1. Mild___________(%) 2. Moderate___________(%) 3. Severe___________(%) |
| 1. Of all your patients with **HAE**, what percentage comes to the consultation with the diagnosis of type I, II and III? | ***Write the %, it should add up to 100%***     1. Type I (C1-INH Deficiency) ________(%) 2. Type II (Deficiency in the functionality of C1-INH, with normal or high levels of C1-INH) ________(%) 3. Type III (in women [X-linked], with normal C1-INH levels and functionality) ________(%) |
| 1. Of all your patients with **HAE**who come to your consultation, what percentage do you diagnose as type I, II and III? | ***Write the %, it should add up to 100%***     1. Type I (C1-INH Deficiency) ________(%) 2. Type II (Deficiency in the functionality of C1-INH, with normal or high levels of C1-INH) ________(%) 3. Type III (in women [X-linked], with normal C1-INH levels and functionality) ________(%) |
| 1. Did you mention what diagnostic methods or tests you use to make an accurate diagnosis of your patients with HAE? | 1. ________________________ 2. ________________________ 3. ________________________ 4. _________________________ |
| 1. Of all your patients with **HAE**, in what percentage are the data presented in the diagnostic tests? | ***Write the %, it should add up to 100%***     1. quantitative levels of C1-INH _____(%) 2. functional levels of C1-INH_____(%) 3. C4 levels + quantitative and functional levels of C1-INH_____(%) |
| 1. Of all your patients with **HAE**on whom you perform genetic tests, what percentage has mutations in…? | 1. mutations in the SERPING1 gene (Types I-II) _____(%) 2. Other mutations _____(%)   Which? _______________________________ |
| 1. Of all your patients with **HAE, what is the % of recurrent**crises /year in general and by age group? | ***Write the %, it should add up to 100%***  Recurrence: ______%   1. < 20 years_________% 2. 20 to 30 years old _______% 3. 30 to 49 years old ______% 4. 50 to 59 years old _______% 5. 60 to 69 years old _____% 6. from 70 to 79 years old_____% 7. > 80 years old_______% |
| 1. On average per year, what percentage of your **HAE patients die for a reason related to the disease**? | ***__________%*** |
| 1. In your patients with **HAE**, and according to the following variables, indicate the frequency per year in which each of these occurs:      \| **HAE** \| **Frequency of Use per year (in-hospital) (relative number or %, as appropriate)** \| \| --- \| --- \| \| **% of total patients receiving emergency care for HAE** \| **_________%** \| \| **# Of admissions for emergency room care** \| 1. **1 (___)** 2. **2 (___)** 3. **3 (___)** 4. **4 (___)** 5. **≥ 5 (___)** \| \| **Percentage of patients who are hospitalized** \| 1. **General____________%** 2. **Suspects __________%** 3. **Likely __________%** 4. **With confirmed diagnosis_______%** \| \| **Average days of hospital stay per event** \| 1. **1 (___)** 2. **2 (___)** 3. **3 (___)** 4. **4 (___)** 5. **≥ 5 (___)** \| \| **# Of patients who are hospitalized in the public and/or private sector in a year** \| **Private sector _________**  **Public sector _________**   - **SSA _________** - **IMSS_________** - **ISSSTE_________** - **OTHERS (PEMEX, SEDENA, SEMAR) _________** \| \| **Number of interconsultations requested per year for diagnosis from other Specialties:**  **(indicate # and what specialties are requested)** \| - ____________(#) - ____________(#) - ____________(#) - ____________(#) \| | |
| 1. In relation to the specific Pharmacological Treatment of its patients with **HAE**, according to the heading      \| **Pharmacological Treatment of the Acute Event** \| \| \| --- \| --- \| \| **Icatibant** \| **%** \| \| **C1-INH concentrate** \| **%** \| \| **Fresh Frozen Plasma** \| **%** \| \| **Ecallantide** \| **%** \| \| **Recombinant C1-INH, Conestat alpha (Ruconest)** \| **%** \| \| **Recombinant human C1 inhibitor (Rhucin)** \| **%** \| \| **Plasma-derived C1-INH (Berinert)** \| **%** \| \| **Plasma-derived C1-INH (Cinryze)** \| **%** \| \| **Others** \| **%** \|        \| **short-term prophylaxis** \| \| \| --- \| --- \| \| **C1-INH concentrate** \| **%** \| \| **Danazol** \| **%** \| \| **Stanozolol** \| **%** \| \| **epsilon amino caproic acid** \| **%** \| \| **Tranexamic Acid** \| **%** \| \| **Plasma-derived C1-INH (Berinert)** \| **%** \| \| **Plasma-derived C1-INH (Cinryze)** \| **%** \| \| **Plasma-derived C1-INH (Haegarda)** \| **%** \| \| **Lanadelumab** \| **%** \| \| **Others** \| **%** \| | |
| 1. According to your experience, where (%) patients with **HAE are cared for:** | ***Write the %, it should add up to 100%***  Private sector _________ (%)  Public sector _________ (%)  SSA _________ (%)  IMSS_________ (%)  ISSSTE_________ (%)  OTHERS (PEMEX, SEDENA, SEMAR) _________ (%) |
| 1. According to your experience, where (%) are patients suspected of **HAE treated:** | ***Write the %, it should add up to 100%***  Private sector _________ (%)  Public sector _________ (%)  SSA _________ (%)  IMSS_________ (%)  ISSSTE_________ (%)  OTHERS (PEMEX, SEDENA, SEMAR) _________ (%) |
| 1. According to your experience, where (%) patients with probable **HAE are cared for:** | ***Write the %, it should add up to 100%***  Private sector _________ (%)  Public sector _________ (%)  SSA _________ (%)  IMSS_________ (%)  ISSSTE_________ (%)  OTHERS (PEMEX, SEDENA, SEMAR) _________ (%) |
| 1. What percentage of your **HAE patients**are referred and how long does it take on average to go from one level to another: | ***Write the %, it should add up to 100%***     1. First level to second level of care______(%)_______(Time) 2. Second level to third level of care______(%)_______(Time) |
| 1. Which specialties frequently refer patients with suspicion or diagnosis of **HAE**? | 1. ***____________________________________*** 2. ***____________________________________*** 3. ***____________________________________*** 4. ***____________________________________*** |
| 1. What percentage of your patients treated with **HAE**are against referrals and how long on average do they take to go from one level to another? | ***Write the %, it should add up to 100%***     1. Third level to second level______(%)_______(Time) 2. Second level to first level______(%)_______(Time) |
| 1. What percentage of your **HAE patients**are referred or counter-referred for follow-up or treatment of another health problem? | 1. Follow-up_________________________(%) 2. Other health problem_____________(%) |
| 1. Do you consider that the treatment of patients with HAE should be always multidisciplinary? | ***Write the %, it should add up to 100%***   1. *Yes______%* 2. *No _______%* |
| 1. Which specialties provide the most support to the **HAE treatment course**? | 1. ***____________________________________*** 2. ***___________________________________*** 3. ***____________________________________*** 4. ***____________________________________*** |
| 1. On average, what is the monthly cost of treatment for a patient with **HAE**? | ***Write the estimated cost:***    **$___________** |
| 1. According to your experience, how do you perceive the quality of life of an adequately treated **HAE patient?** | ***Mark the appropriate answer:***  Bad ____   Good ____   Regular ____  Excellent ____ |
