# Supplementary material for: Real-world experience of hereditary angioedema (HAE) in Mexico: A mixed-methods approach to describe epidemiology, diagnosis, and treatment patterns
Source: World Allergy Organ J. 2023 Sep 13;16(9):100812. doi: 10.1016/j.waojou.2023.100812 (PMC10506135; doi:10.1016/j.waojou.2023.100812)

**Supplement 4**

**Diagram. Estimation Process. Prevalence, Type HAE, Gender and Age Group Distribution. México, 2019.**


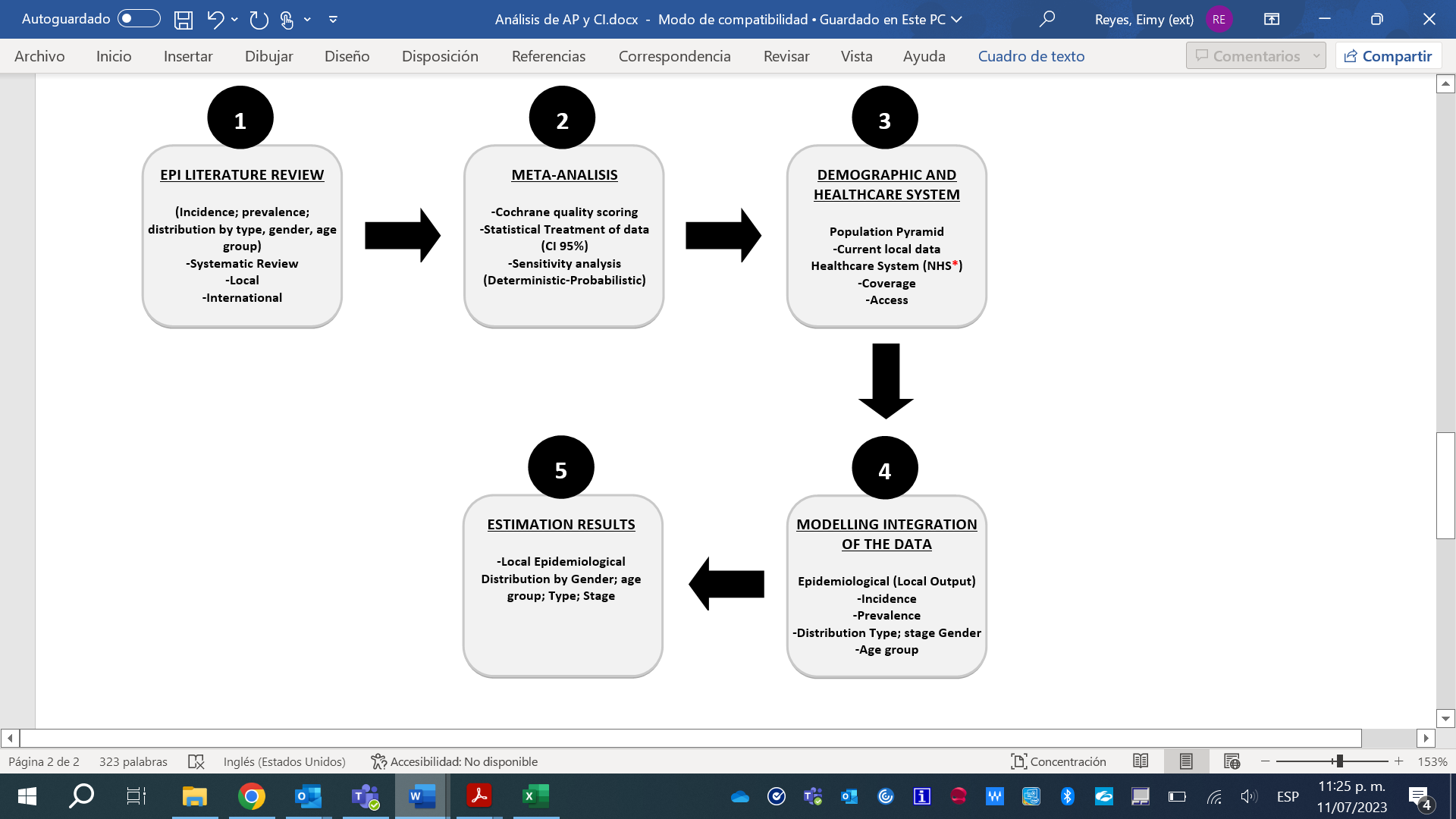


**Diagram. Estimation Process. Cases Seen and Treated. Type 1 HAE. AEP survey. Clinical Profile. Treatment. Mexico, 2019.**


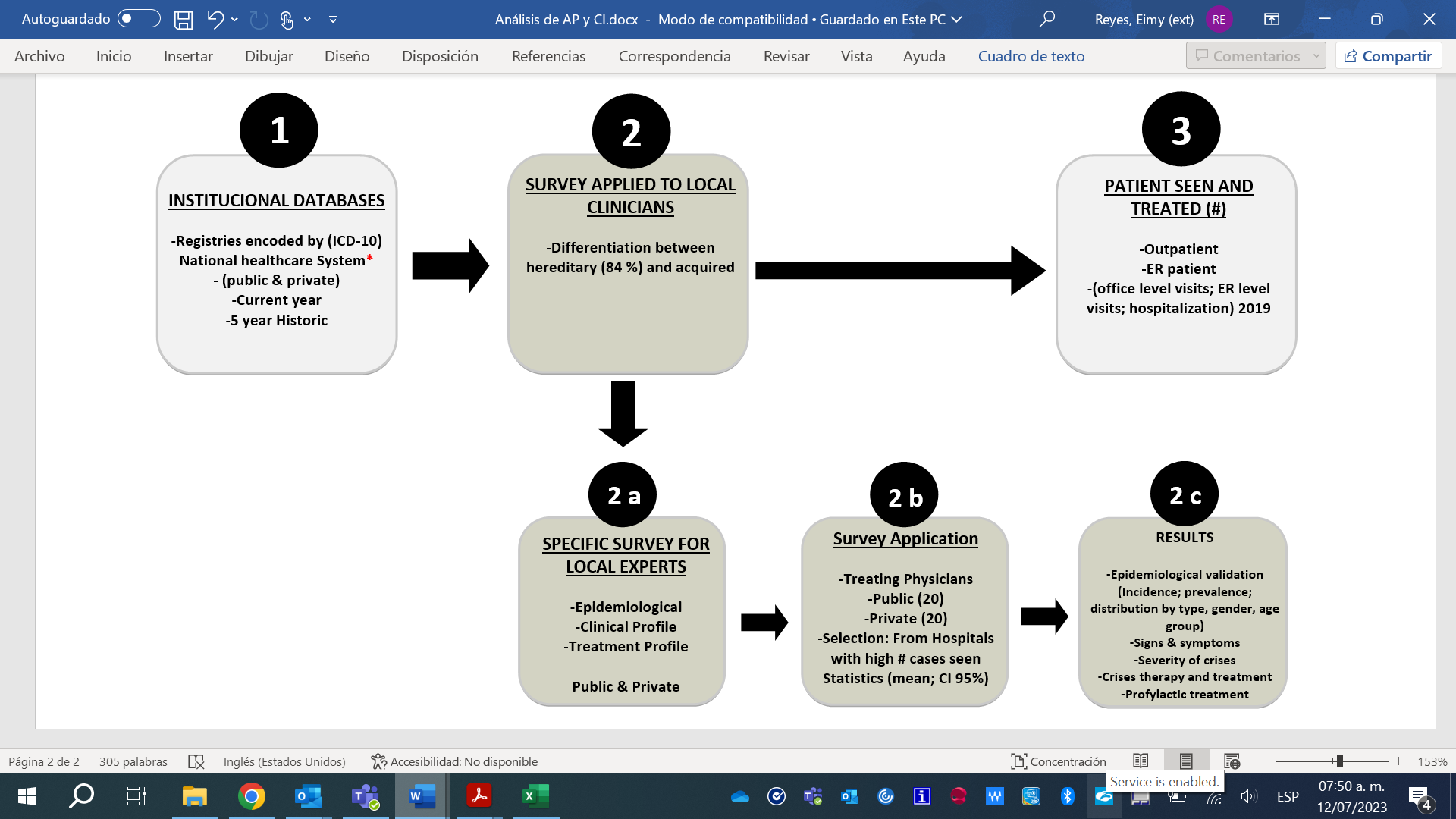

Supplement: Multimedia component 4 [file mmc4.docx]
